# Genome characterization of cluster EK1 bacteriophages JessellCookie and MiamiPanther

**DOI:** 10.1128/mra.01242-25

**Published:** 2026-03-05

**Authors:** Gabriela Barredo, Isabella Bauchiero, Denis Cabellero, Zachyra Cortes, Liam De Armas, Lucas Gonzalez, Thalia Oliveros, Martina Podesta Quistapace, Odette Pulido, Lara Becerra Reymundo, Xavier S. Purroy, Betty R. Sierra, Patricia Waikel, Jaime Mayoral

**Affiliations:** 1 Department of Biological Sciences, Florida International University 5450, Miami, Florida, USA; Portland State University, Portland, Oregon, USA

**Keywords:** Podovirus, *Microbacterium*, genome analysis, bacteriophages

## Abstract

Bacteriophages JessellCookie and MiamiPanther were isolated from environmental samples collected in Miami and Miami Beach, FL, respectively, using *Microbacterium foliorum* as host. Both phages have a podoviral morphology and are grouped in the EK1 subcluster based on gene content similarity.

## ANNOUNCEMENT

*Microbacterium foliorum* NRRL B-24224 is a yellow-pigmented, gram-positive rod-shaped bacterium (1) that is easily cultured and thereby convenient as a host for the isolation and characterization of novel bacteriophages. Such exploration is invaluable to advancing our understanding of bacteriophage diversity, evolution, and gene function.

**Fig 1:**
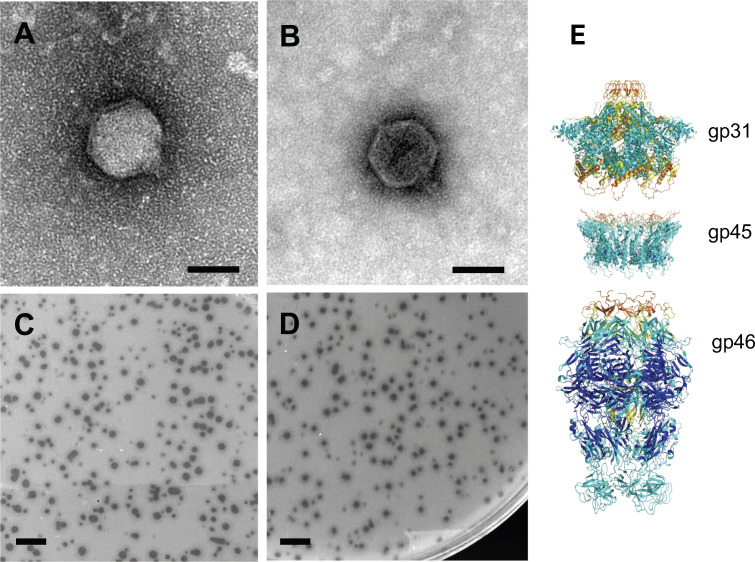
Transmission electron microscopy, plaques, and putative tail tip structures. Transmission electron microscopy of (**A**) JessellCookie and (**B**) MiamiPanther showing a podoviral morphology, scale bars = 50 nm. Plaque assays show phages forming clear plaques with small halos for both (**C**) JessellCookie and (**D**) MiamiPanther, scale bars 5 mm. (**E**) JessellCookie structural predictions of genes gp31, gp45, and gp46 using AlphaFold and FoldSeek with suggested tentative functions: portal, adaptor, and nozzle proteins. Electron microscopy micrographs were taken using a Hitachi HT7800 120 kV TEM with an AMT Nanosprint15 B digital camera.

JessellCookie and MiamiPanther were isolated from topsoil samples collected in Miami and Miami Beach, FL, respectively (GPS coordinates, Table 1), in September 2023. Soil samples were suspended in PYCa liquid medium (2), centrifuged at 2,000 *g,* and filtered through a 0.22 μm filter. Phages capable of infecting *Microbacterium foliorum* were enriched by inoculating the filtrate with *M. foliorum* strain NRRL B-24224 in PYCa liquid medium and incubating at 30°C for 48 h. Enriched samples were filtered, plated in PYCa top agar with *M. foliorum*, and incubated at 30°C for 24 h. Individual plaques were picked and purified through two rounds of plating. Lysates were collected after flooding webbed plates with Phage Buffer for 2 h. Plaque characteristics are provided in Table 1 and Fig. 1. TEM revealed a podoviral morphology for both bacteriophages (Fig. 1), and viral morphometrics are shown in Table 1.

**TABLE 1:** Sample locations and features of JessellCookie and MiamiPanther EK1 subcluster actinobacteriophages

Phage	JessellCookie	MiamiPanther
Phage Collection Coordinates	26.2957 N, 80.18223 W	25.78789 N, 80.1291 W
Plaque Size (mm) (*N*=10)	0.91 ± 0.23	0.76 ± 0.17
Capsid Diameter (nm) (*N*=3)	72.90 ± 1.57	62.01 ± 1.50
Number of 100-based Sequencing Reads	2,061,415	2,508,311
Shotgun Sequencing Coverage	3,153	4,213
Genome Size (bp)	53,947	53,944
GC Content (%)	56.7%	56.7%
Genome End Type	circularly permuted	circularly permuted
Number of Putative Protein-Coding Genes	55	55
Number of Membrane Proteins	5	5
Number of Genes Assigned a Putative Function	16	16
Number of tRNAs	0	0

DNA was extracted from phage lysates using the *Quick*-DNA Viral Kit (Zymo). DNA was prepared for sequencing using the NEB Ultra II FS Kit and sequenced using the Illumina NextSeq 1000 (XLEAP-P1 kit), generating 100-base single-end reads. Raw reads were trimmed with cutadapt 4.7 (using the option: –nextseq-trim 30) and filtered with skewer 0.2.2 (using the options: -q 20 -Q 30 -n -l 50) prior to assembly (3); genome ends were assessed as previously described (3). Reads were then assembled using Newbler v2.9 and genomes checked for completeness using Consed (4, 5). Sequencing data and genome characteristics for the two phages are described in Table 1.

The genomes were annotated using DNA Master v.4.2.1.11 , Glimmer v.3.02 (6), Genemark v.3.25 (7), Starterator v600 (http://phages.wustl.edu/starterator), Phamerator v.594 (8), TMHMM v2.0 (9), BLASTp (10) using the NCBI non-redundant database, HHpred (11) using PDB_mmCIF70, Pfam-A_v37, UniProt-SwissProt-viral70, and NCBI_Conserved Domains databases, PECAAN v.20241104 (https://discover.kbrinsgd.org), Aragorn v2.41 (12) and tRNAscanSE v2.0 (13). Default settings were used for all software unless otherwise specified.

JessellCookie’s genome contains 53,947 bp and 55 predicted genes. ORFs are encoded on one strand for half of the genome, and on the other strand for the other half. Putative functions were assigned to 16 genes. No programmed translational shift or tRNAs were identified (Table 1). JessellCookie’s gene gp31 was annotated as a portal protein. AlphaFold (14) and FoldSeek (15) structural predictions identified gp45 and gp46 as the adaptor and nozzle proteins (Fig. 1).

MiamiPanther’s 53,944 bp genome is similar to JessellCookie in gene content and genome structure. They share the same number and function of putative genes (Table 1). They differ by 860 bp, sharing a 98.4% identity and 96.5% gene content similarity (GCS). MiamiPanther and JessellCookie were assigned to cluster EK1 based on a GCS of at least 35% to phages in the Actinobacteriophage database, Phagesdb ( https://phagesdb.org/) (5, 16). No immunity repressor or integrase was identified for either phage, suggesting they are unlikely to establish lysogeny.

## Data Availability

JessellCookie is available at GenBank accession no. PV876967 and Sequence Read Archive (SRA) no. SRR33237356. MiamiPanther is available at GenBank accession No. PV876985 and Sequence Read Archive (SRA) no. SRR33237352.

## References

[1] Gneiding K, Frodl R, Funke G. 2008. Identities of Microbacterium spp. encountered in human clinical specimens. J Clin Microbiol 46:3646–3652. doi:10.1128/JCM.01202-08 18799696 PMC2576590

[1] SEA-PHAGES. 2025. Phage discovery guide. Available from: https://discoveryguide.seaphages.org/

[3] Russell DA. 2018. Sequencing, assembling, and finishing complete bacteriophage genomes. Methods Mol Biol 1681:109–125. doi:10.1007/978-1-4939-7343-9_9 29134591

[4] Gordon D, Abajian C, Green P. 1998. Consed: a graphical tool for sequence finishing. Genome Res 8:195–202. doi:10.1101/gr.8.3.195 9521923

[5] Russell DA, Hatfull GF. 2017. PhagesDB: the actinobacteriophage database. Bioinformatics 33:784–786. doi:10.1093/bioinformatics/btw711 28365761 PMC5860397

[6] Delcher AL, Bratke KA, Powers EC, Salzberg SL. 2007. Identifying bacterial genes and endosymbiont DNA with Glimmer. Bioinformatics 23:673–679. doi:10.1093/bioinformatics/btm009 17237039 PMC2387122

[7] Besemer J, Borodovsky M. 2005. GeneMark: web software for gene finding in prokaryotes, eukaryotes and viruses. Nucleic Acids Res 33:W451–4. doi:10.1093/nar/gki487 15980510 PMC1160247

[8] Cresawn SG, Bogel M, Day N, Jacobs-Sera D, Hendrix RW, Hatfull GF. 2011. Phamerator: a bioinformatic tool for comparative bacteriophage genomics. BMC Bioinformatics 12:395. doi:10.1186/1471-2105-12-395 21991981 PMC3233612

[9] Krogh A, Larsson B, von Heijne G, Sonnhammer EL. 2001. Predicting transmembrane protein topology with a hidden Markov model: application to complete genomes. J Mol Biol 305:567–580. doi:10.1006/jmbi.2000.4315 11152613

[10] Altschul SF, Gish W, Miller W, Myers EW, Lipman DJ. 1990. Basic local alignment search tool. J Mol Biol 215:403–410. doi:10.1016/S0022-2836(05)80360-2 2231712

[11] Söding J, Biegert A, Lupas AN. 2005. The HHpred interactive server for protein homology detection and structure prediction. Nucleic Acids Res 33:W244–8. doi:10.1093/nar/gki408 15980461 PMC1160169

[12] Laslett D, Canback B. 2004. ARAGORN, a program to detect tRNA genes and tmRNA genes in nucleotide sequences. Nucleic Acids Res 32:11–16. doi:10.1093/nar/gkh152 14704338 PMC373265

[13] Lowe TM, Eddy SR. 1997. tRNAscan-SE: a program for improved detection of transfer RNA genes in genomic sequence. Nucleic Acids Res 25:955–964. doi:10.1093/nar/25.5.955 9023104 PMC146525

[14] Jumper J, Evans R, Pritzel A, Green T, Figurnov M, Ronneberger O, Tunyasuvunakool K, Bates R, Žídek A, Potapenko A, et al.. 2021. Highly accurate protein structure prediction with AlphaFold. Nature 596:583–589. doi:10.1038/s41586-021-03819-2 34265844 PMC8371605

[15] van Kempen M, Kim SS, Tumescheit C, Mirdita M, Lee J, Gilchrist CLM, Söding J, Steinegger M. 2024. Fast and accurate protein structure search with Foldseek. Nat Biotechnol 42:243–246. doi:10.1038/s41587-023-01773-0 37156916 PMC10869269

[16] Pope WH, Mavrich TN, Garlena RA, Guerrero-Bustamante CA, Jacobs-Sera D, Montgomery MT, Russell DA, Warner MH, Hatfull GF, Science Education Alliance-Phage Hunters Advancing Genomics and Evolutionary Science (SEA-PHAGES). 2017. Bacteriophages of Gordonia spp. display a spectrum of diversity and genetic relationships. mBio 8. doi:10.1128/mBio.01069-17 PMC5559632 28811342

